# Characterization of Six Ampeloviruses Infecting Pineapple in Reunion Island Using a Combination of High-Throughput Sequencing Approaches

**DOI:** 10.3390/v16071146

**Published:** 2024-07-16

**Authors:** Delphine Massé, Thierry Candresse, Denis Filloux, Sébastien Massart, Nathalie Cassam, Bruno Hostachy, Armelle Marais, Emmanuel Fernandez, Philippe Roumagnac, Eric Verdin, Pierre-Yves Teycheney, Jean-Michel Lett, Pierre Lefeuvre

**Affiliations:** 1 ANSES—LSV RAPT, F-97410 St. Pierre, La Réunion, France; nathalie.cassam@anses.fr (N.C.); bruno.hostachy@anses.fr (B.H.); 2 UMR PVBMT, Université de La Réunion, F-97410 St. Pierre, La Réunion, France; 3 INRAe, UMR 1332 Biologie du Fruit et Pathologie, Université Bordeaux, CS20032, F-33882 Villenave d’Ornon, France; thierry.candresse@inrae.fr (T.C.); armelle.marais-colombel@inrae.fr (A.M.); 4 CIRAD, UMR PHIM, F-34090 Montpellier, France; denis.filloux@cirad.fr (D.F.); emmanuel.fernandez@cirad.fr (E.F.); philippe.roumagnac@cirad.fr (P.R.); 5 PHIM Plant Health Institute, Université Montpellier, CIRAD, INRAE, Institut Agro, IRD, F-34090 Montpellier, France; 6 Plant Pathology Laboratory, Gembloux Agro-Bio Tech, University of Liège, 5030 Gembloux, Belgium; sebastien.massart@uliege.be; 7 INRAe, UR407 Unité de Pathologie Végétale, CS 60094, F-84140 Montfavet, France; eric.verdin@inrae.fr; 8 CIRAD, UMR PVBMT, F-97410 St. Pierre, La Réunion, France; pierre-yves.teycheney@cirad.fr (P.-Y.T.); jean-michel.lett@cirad.fr (J.-M.L.); pierre.lefeuvre@cirad.fr (P.L.)

**Keywords:** *Ananas comosus*, pineapple mealybug wilt-associated viruses, ampelovirus, high-throughput sequencing, short and long reads, molecular diversity

## Abstract

The cultivation of pineapple (*Ananas comosus*) is threatened worldwide by mealybug wilt disease of pineapple (MWP), whose etiology is not yet fully elucidated. In this study, we characterized pineapple mealybug wilt-associated ampeloviruses (PMWaVs, family *Closteroviridae*) from a diseased pineapple plant collected from Reunion Island, using a high-throughput sequencing approach combining Illumina short reads and Nanopore long reads. Reads co-assembly resulted in complete or near-complete genomes for six distinct ampeloviruses, including the first complete genome of pineapple mealybug wilt-associated virus 5 (PMWaV5) and that of a new species tentatively named pineapple mealybug wilt-associated virus 7 (PMWaV7). Short reads data provided high genome coverage and sequencing depths for all six viral genomes, contrary to long reads data. The 5′ and 3′ ends of the genome for most of the six ampeloviruses could be recovered from long reads, providing an alternative to RACE-PCRs. Phylogenetic analyses did not unveil any geographic structuring of the diversity of PMWaV1, PMWaV2 and PMWaV3 isolates, supporting the current hypothesis that PMWaVs were mainly spread by human activity and vegetative propagation.

## 1. Introduction

Pineapple (*Ananas comosus* L. [Merr.]) is a tropical plant in the family *Bromeliaceae*. It is grown extensively throughout the tropics and subtropics for its edible fruits. In 2021, worldwide pineapple production was estimated at 28.6 million metric tons, making it the third largest tropical fruit production [1]. Among the many pests and diseases that hamper the cultivation of pineapple, mealybug wilt of pineapple (MWP) is considered the most important and complex [2]. Typical symptoms of MWP include reddening and/or yellowing of the leaves, downward curling of margin tips, leaf-tip dieback, wilting and root decay [3,4,5]. Since its first description in Hawaii in 1910 [6], MWP has been described in most pineapple growing areas and reported to cause yield reductions of up to 55% [7,8].

The involvement of a virus in the etiology of MWP was first supported by immunological evidence [9]. Long, flexuous, rod-shaped virus particles and high molecular weight double stranded RNAs (i.e., nucleic acid forms originating mostly from viruses) were later observed in infected plants [10,11], and associated viruses were assigned to genus *Ampelovirus* [2,12] in the family *Closteroviridae* [13]. Ampeloviruses have single stranded positive-sense RNA genomes ranging in size between 13.0 and 18.5 kb, with 7 to 12 open reading frames (ORFs) [14]. They are distributed in two subgroups (I and II) depending on genome organisation and phylogenetic relationships [15,16]. Thirteen species are currently assigned to the *Ampelovirus* genus, including *Ampelovirus unananas*, *Ampelovirus duananas* and *Ampelovirus triananas*, whose exemplar isolates are pineapple mealybug wilt-associated virus 1 (PMWaV1), PMWaV2 and PMWaV3, respectively [15]. Additional ampeloviruses were described in pineapple but not yet included in the taxonomy by the International Committee on Taxonomy of Viruses (ICTV): PMWaV4, which was later reassigned as a strain of PMWaV1 [17]; PMWaV5 [7,17], for which only a partial genome sequence is available; and more recently PMWaV6, for which a complete genome sequence was obtained using high-throughput sequencing (HTS) [18]. Ampeloviruses are transmitted by mealybugs (*Pseudococcidae*) and scale insects (*Coccidae*), in a semi-persistent mode [14,19]. There is no evidence of seed or mechanical transmission. Long distance dissemination primarily occurs through the exchange of infected propagation materials and germplasm [20].

The etiology of MWP has not yet been fully elucidated. Biotic and abiotic factors, such as environmental factors, the presence of mealybugs and co-infections with distinct ampeloviruses, and/or with viruses from other genera may be involved. In Hawaii, only pineapple plants infected by PMWaV2 and infested with mealybugs were reported to display MWP symptoms [21]. However, another study conducted in Australia suggested that the presence of PMWaV1 and PMWaV3 together with other viruses such as badnaviruses, was involved in the etiology of MWP [7]. Additional viruses infecting pineapple were recently characterized [22,23,24,25], further increasing the number of virus combinations possibly involved in the etiology of MWP, and challenging serological or PCR-based viral diagnostics. HTS-based approaches, which have enabled the discovery and detection of new viruses and viral strains in a large range of plants [26,27,28], have been successfully applied to the characterization of several ampeloviruses infecting pineapple [18,29] and have a definite potential for comprehensive diagnosis of pineapple-associated viruses.

The current diversity of pineapple viruses and the still-unresolved etiology of MWP argue for a better characterization of the pineapple virome. Hence, we compared and assessed two different HTS strategies for the characterization of ampelovirus genomes using total RNA extracted from the leaves of a pineapple plant showing MWP symptoms. The first strategy is based on cDNA synthesis from ribodepleted total RNAs and Illumina short reads sequencing, whereas the second is based on cDNA synthesis from poly-A tailed RNAs and Nanopore long read sequencing. Using the combination of these two methods, we obtained near complete genome sequences of PMWaV2, PMWaV3, PMWaV5, and PMWaV6, and complete genome sequences of PMWaV1 and a putative new ampelovirus tentatively named pineapple mealybug wilt-associated virus 7 (PMWaV7). Phylogenetic analyses of PMWaV1, PMWaV2 and PMWaV3 suggested that recurrent exchanges of infected germplasm promoted the worldwide dissemination of these viruses.

## 2. Materials and Methods

### 2.1. Plant Material

A plant of cultivar “Queen Victoria” showing reddening and leaf tip dieback symptoms was collected in 2016 from Cirad’s experimental station at Bassin Plat in Saint Pierre (Reunion Island, France). Two full leaves from this plant (referred to as sample 16-1) were cut into ~0.5 cm^2^ pieces and stored at −80 °C until further use.

### 2.2. RNA Extraction

Total RNAs were extracted from the 16-1 leaf sample using the RNeasy Plant Mini Kit (QIAGEN, Hilden, Germany) according to the manufacturer’s instructions, including an optional DNAse treatment (DNAse I, Thermo Fisher Scientific, Waltham, MA, USA). The suitability of extracted RNAs quality for HTS sequencing was assessed by measuring the A260/280 (~2.0) and A260/230 (2.0 to 2.2) ratios using a NanoVue spectrophotometer (Cytiva, Washington, DC, USA) and the RNA Integrity Number (RIN, >6.0) value using an Agilent TapeStation and an RNA 6000 Pico chip (Agilent Technologies, Santa Clara, CA, USA). Extracted RNAs were quantified using a Qubit Fluorometer 2.0 (Qubit RNA Broad Range Assay Kit, Thermo Fisher Scientific, Waltham, MA, USA). Total RNA samples were stored at −80 °C until further use.

### 2.3. High-Throughput Sequencing

Two HTS approaches were used in the study. In the first approach (hereafter referred to as “Illumina short reads approach”), two Illumina sequencing runs were performed. For both runs, total RNAs were first ribodepleted prior to cDNA synthesis and sequencing. The first run was performed according to Marais et al. [30]. Briefly, ribosomal RNAs were removed from the total RNAs using a RiboMinus Plant Kit (Invitrogen, Fisher Scientific, Illkirch, France). A cDNA library was generated using the Illumina TruSeq Stranded total RNA library prep kit (Illumina, San Diego, CA, USA) and sequenced on an Illumina NextSeq500 sequencer (2 × 150 bp) (GIGA-Genomics Facility, Université de Liège, Liège, Belgium). For the second run, libraries preparation and sequencing were performed by Genewiz (now Azenta Life Sciences, South Plainfield, NJ, USA) using the Ribozero plant rRNA depletion kit and the TruSeq stranded total RNA library Prep kit (Illumina, San Diego, CA, USA), followed with a paired-ends 2 × 150 bp run on an Illumina NovaSeq sequencer.

In the second approach, hereafter referred to as “Nanopore long reads approach”, nanopore sequencing was performed using the MinION device (Mk1B, Oxford Nanopore Technologies, Oxford, UK). For this, a cDNA library was generated from the total RNAs using poly(dT) primers, with the cDNA-PCR Barcoding kit SQK-PCB109 (Oxford Nanopore Technologies, Oxford, UK) according to the manufacturer’s instructions, except that Sera-Mag Select Size Selection beads (Cytiva, Washington, DC, USA) were used for the nucleic acid purification step. Two distinct sequencing runs were performed. In the first one, the RNA sample was treated in quadruplicate using distinct barcodes. A cDNA library with equimolar concentrations of products from each replicate was produced. In the second run, a single cDNA library was prepared from the RNA sample. In both runs, libraries were loaded on FLO-MIN 106D R9.4.1 flowcells and sequencing was performed using a Mk1B MinION device and monitored using the MinKNOW v21.11.8 software (ONT) for 16 and 28 h, respectively.

### 2.4. Data Analysis and Assembly of Viral Genomes

Sequencing data were uploaded on the Galaxy instance of the Migale bioinformatics server (https://migale.inrae.fr) and analysed using a homemade workflow described below. Demultiplexing and quality control of short reads were performed using Trimmomatic v0.38.1 [31]. After adapters and tags removal using default settings, reads were quality trimmed using a minimum quality score of 25 and a minimum read length of 80 nt. For long reads, highly accurate base calling was performed using the Guppy v6.0.1 software (ONT), and reads that passed the default quality threshold (mean read PHRED score of 7) were processed with PoreChop v0.2.4 [32] for adapters removal and demultiplexing when needed. Reads shorter than 80 nt were removed using NanoFilt v0.1.0 [33].

Hybrid de novo assembly of short and long reads was performed using SPAdes v3.13 [34] on the SouthGreen bioinformatic platform [35]. Then, viral contigs were identified using BLASTn and BLASTx searches against the reference viral sequences database retrieved from GenBank in April 2022 (v211). When possible, viral contigs were merged using the EGAssembler [36] before being polished using Pilon v1.23 [37] after mapping back short reads using the BWA-MEM algorithm (BWA v0.7017.1) [38]. Each set of trimmed and demultiplexed reads was individually mapped against polished viral contig references using BWA-MEM for short reads and Minimap2 v2.17 [39] for long reads, respectively. After discarding non-primary alignments, mapping statistics were obtained using Samtools v1.9 [40]. Subsamplings of the mapped reads were performed in order to estimate the coverage of viral contigs as a function of total reads number. To this end, we evaluated the number of reads mapped per position for sets of decreasing sequencing efforts with 100 random replicates per set. Sequencing depth (the number of times a position was covered with a read) and breadth of coverage (the proportion of the genome that is covered with reads) were computed for each subsample.

### 2.5. Detection of PMWaVs by RT-PCR

RT-PCR diagnosis was performed in order to confirm the presence of the viruses detected by HTS. The detection of PMWaV1, PMWaV2 and PMWaV3 was performed by a new multiplex RT-PCR using virus-specific primers [2,7] (Supplementary Table S1). Additional simplex RT-PCRs were carried out to detect PMWaV5, PMWaV6 and PMWaV7 using virus-specific primers (Supplementary Table S1) designed from available complete viral genome sequences.

Reactions were performed using the One-Step RT-PCR kit (QIAGEN, Hilden, Germany) in a Veriti 96-Well Fast Thermal Cycler (Applied Biosystems™, Thermo Fisher Scientific, Waltham, MA, USA). For the multiplex detection of PMWaV1, PMWaV2 and PMWaV3, the reaction mix contained 2.5 µL of 5X RT-PCR Buffer, 0.5 µL of 10 mM dNTPs, 1.25 µL of a primer mix containing PMWaV1 and PMWaV3 primers at a concentration of 10 mM, 2 µL of a primer mix containing the PMWaV2 specific primers at a concentration of 10 mM, 2 µL of RT-Taq DNA polymerase mix, 2 µL of total plant RNA extracts and nuclease-free water for a total reaction volume of 25 µL. For the simplex RT-PCR detection of PMWaV5, PMWaV6 or PMWaV7, the reaction mix contained 5 µL of 5X RT-PCR buffer, 1 µL of 10 mM dNTPs, 1.5 µL of a primer mix containing each specific primer at a concentration of 10 mM, 1 µL of RT-Taq DNA polymerase mix, 2 µL of total plant RNA extracts and nuclease-free water for a total reaction volume of 25 µL. RT-PCR conditions were a reverse-transcription step of 30 min at 50 °C followed by an initial denaturation step of 15 min at 95 °C and 35 cycles of 30 s at 94 °C, 30 s at 58 °C (for PMWaV1, PMWaV2 and PMWaV3) or 60 °C (for PMWaV5, PMWaV6, and PMWaV7), and 1 min at 72 °C, then a final extension of 10 min at 72 °C. Amplification products were analyzed by electrophoresis on 2.5% agarose gels in a Tris-acetate-EDTA (TAE) buffer and visualized under UV light following staining with ethidium bromide.

### 2.6. RACE PCR

Rapid amplification of cDNA 3′-ends (3′-RACE) and 5′-ends (5′-RACE) experiments were performed for viruses for which genomic 5′ and 3′ ends were missing from the assemblies of HTS reads. The SMARTer RACE 5′/3′ Kit (Takara, Beijing, China) was used following the manufacturer’s instructions. cDNAs were amplified by PCR using the universal primer A mix provided by the manufacturer and a virus-specific primer designed from the assembled genome sequence (Supplementary Table S2). The resulting PCR amplicons were analyzed by electrophoresis as described above. Bands corresponding to amplification products of the expected sizes were excised from the gels under UV light, purified using the Nucleospin PCR and Gel Purification Kit (Macherey-Nagel, Düren, Germany) and ligated into the CloneJet PCR cloning vector (Thermo Scientific, Waltham, MA, USA). Inserts of the recombinant vectors were sequenced by standard Sanger sequencing (Macrogen Inc., Seoul, Republic of Korea) using a primer walking approach.

### 2.7. Search for Recombination and Phylogenetic Analyses

Sequence alignments were performed using MAFFT v7.450 in Geneious Prime soft v2021.1 [41]. Inter-species recombination signals were searched using the RDP [42], GENECONV [43], BOOTSCAN [44], MAXCHI [45], CHIMERA [46], SISCAN [47] and 3SEQ [48] methods implemented in the RDP5 program [49], using default settings. Recombination events were considered significant when detected (*p*-value < 0.05) by at least three different recombination detection methods.

For phylogenetic analyses, subsets of nucleotide sequences encoding viral RNA-dependant RNA polymerase (RdRp), heat shock protein 70 homolog (HSP70h) and coat protein (CP) sequences were obtained from the complete genome sequences, and conceptually translated into amino acid sequences. Homologous sequences of beet yellows virus (genus *Closterovirus*) were used as outgroups. Sequences alignments were performed using MAFFT v7.450 [41]. Maximum likelihood phylogenetic reconstruction was performed using FastTree v2.1.11 [50]. The LG substitution model with gamma-distributed rate among sites was used and the Shimodaira–Hasegawa-like test (SH-like) for branch support (1000 replicates) was performed.

## 3. Results

### 3.1. Identification of Known and Novel Ampeloviruses

HTS performed on sample 16-1 generated 136.9 M paired reads (representing ~20.1 Gb) and 4.9 M reads (representing ~3.3 Gb) from the Illumina short reads and Nanopore long reads approaches, respectively. After trimming, the size of long reads ranged from 80 to 8053 nt with a mean size of 615 nt, and that of short reads ranged from 80 to 150 nt with a mean size of 122 nt. A total of 97,021 contigs (ranging in size between 80 and 56,804 nt) was obtained from de novo assembly performed on both short and long reads using the hybrid assembly approach. After similarity searches against viral databases, 183 of these contigs were identified as viral sequences. After aligning and merging these 183 viral contigs, a second similarity search was performed against viral databases using BLASTn and revealed the presence of six supercontigs (referred to as Contig-A to Contig-F) ranging in size between ~13 and 18 kbp, with similarities to viruses from the genus *Ampelovirus* (Table 1), especially PMWaV1, PMWaV2, PMWaV3 and PMWaV6 for which similarities ranged between 79% and 99% nucleotide identity (E-values of 0) (Table 1).

Other contigs with similarities with viruses from genera *Vitivirus* (family *Betaflexiviridae* [25]), *Sadwavirus* (family *Secoviridae*) [22,23,24]) and *Badnavirus* (family *Caulimoviridae* [21]) genera, were also obtained (Supplementary Table S3).

The presence of a strand-switching primer (SSP) sequence and poly-A tail within some of the long reads allowed the identification of the putative 5′-ends for five out of the six assembled genomes (except for Contig-D) and the putative 3′-ends of Contig-A and Contig-F. Using RACE PCR and custom primers (Supplementary Table S2) designed from the sequences of the contigs, the 5′-ends of Contig-D, Contig-E and Contig-F and the 3′-end of Contig-F were confirmed. Our attempts to obtain the sequences of 3′-ends of Contig-B, Contig-C, Contig-D and Contig-E remained unsuccessful. The presence of the six identified ampeloviruses in sample 16-1 was confirmed using RT-PCR assays performed with virus-specific primers (Supplementary Table S1).

### 3.2. Species Identification and Genome Organization

**Figure 1:**
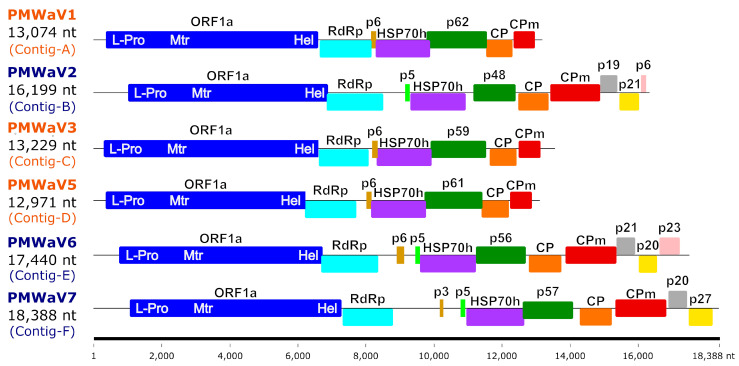
Genomic organization of the six ampelovirus isolates whose genomes were sequenced in this work. ORFs are represented as coloured boxes. Size scale (in nucleotides) is provided at the bottom of the figure. The acronyms of ampeloviruses from subgroup I and subgroup II are coloured in blue and orange, respectively. Abbreviations are as follows: L-Pro, leader papain-like protease; Mtr, methyltransferase; Hel, helicase; RdRp, RNA-dependent RNA polymerase; HSP70h, heat-shock protein 70 homolog; CP, coat protein; CPm, minor coat protein; other ORFs are named according to the weight in kDa of the protein they encode, preceded with the letter “p” for protein.

The sizes of the six assembled ampelovirus genomes, including the 5′ and 3′ ends, ranged between 12,971 and 18,388 nt (Table 1 and Figure 1). These genomes shared a common organisation typical of either ampelovirus subgroup I (with seven ORFs) or subgroup II (with 10 or 11 ORFs) (Figure 1 and Supplementary Table S4).

The RdRp, HSP70h and CP amino acid sequences conceptually translated from the six assembled ampelovirus genomes were used in phylogenetic analyses to assign these genomes to existing or new ampelovirus species, following the current ICTV criteria for species demarcation in genus *Ampelovirus* of less than 75% amino acid (aa) identity [14]. RdRps, HSP70hs, and CPs encoded by Contigs-A, -B, -C and -E displayed 86.4 to 99.2% identity with homologous proteins of previously characterized isolates of PMWaV1, PMWaV2, PMWaV3, and PMWaV6, respectively (Table 2), providing evidence that Contigs-A, -B, -C and -E can be safely assigned to isolates of PMWaV1, PMWaV2, PMWaV3 and PMWaV6, respectively, named hereafter PMWaV1-RUN, PMWaV2-RUN, PMWaV3-RUN and PMWaV6-RUN, respectively. The genomes of the four PMWaV6 isolates from Hawaii for which genome sequences are publicly available, and that of PMWaV6-RUN varied in size due to insertion/deletion, notably between the ORFs encoding the RdRp and p6. The genome of PMWaV6-RUN (17,355 nt) displayed a 470 nt deletion similar to that of isolate 6/S1-1 from Hawaii. None of the other three isolates of PMWaV6 described in Hawaii (S1, S2-1 and S3-1), whose sizes range from 17,225 to 18,213 nt, displayed this deletion.

The RdRp and HSP70h conceptually translated from the complete genome sequence corresponding to Contig-D shared 85.7% and 91.8% aa identity, respectively, with their counterparts encoded by the only publicly available and partial PMWaV5 genome sequence (EF467920) [6], providing a strong indication that Contig-D corresponds to the complete genome of a Reunion isolate of PMWaV5 tentatively named PMWaV5-RUN (OQ850040), making it the first complete genome of a PMWaV5 isolate. Sequence comparisons of the CP sequence of this isolate with that of the partially sequenced genome could not be carried out, since this partial sequence (EF467920) does not encompass the ORF encoding the CP. The genome of PMWaV5-RUN displays the typical organisation of subgroup II ampeloviruses, with seven putative ORFs (Supplementary Table S4). ORF1a encodes a putative polyprotein of 226.8 kDa with the domains of a papain-like protease (L-Pro), a methyltransferase (Mtr) and a helicase (Hel) involved in replication [51]. ORF1b encodes a putative 52.3 kDa protein with the conserved motifs of an RNA-dependent RNA polymerase (RdRp). ORF2 encodes a putative movement protein of 5.7 kDa [52]. ORF3, ORF4, ORF5 and ORF6 encode a putative HSP70h (58.4 kDa), p61 (61.2 kDa), CP (30 kDa) and coat protein minor (CPm; 24.3 kDa), respectively.

**Figure 2:**
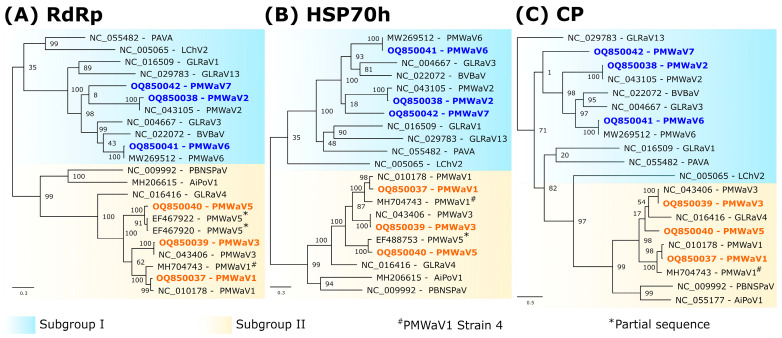
Maximum-likelihood phylogenetic trees showing the placement of isolates of pineapple mealybug wilt-associated viruses (PMWaV1 to 7) within genus *Ampelovirus*, using the amino acid sequences of the RdRp (**A**), HSP70h (**B**) and CP (**C**). Sequences obtained during this study are indicated in bold. Values at nodes represent bootstrap supports of the branches to their left. The scale bars represents the number of substitutions per site. Beet yellows virus (BYV, *Closterovirus*) was used as an outgroup and solely the *Ampelovirus* clade was presented for convenience. *Ampelovirus* subgroups I and II are shown in blue and yellow, respectively. Partial sequences are indicated with asterisks. PMWaV4 was recently classified as a strain of PMWaV1 and is indicated with a hashtag. For virus acronyms, see Supplementary Table S5.

The RdRp and HSP70h conceptually translated from the complete genome sequence corresponding to Contig-F (18,388 nt) were most closely related to those of grapevine leafroll-associated virus 3 (GRLaV3, NC_004667), with 43.0% and 41.2% aa identities, respectively, whereas the CP was most closely related to that of the blackberry vein banding-associated virus (BVBaV, NC_022072), with 34.3% aa identity (Table 2). Therefore, following the criterion for the demarcation of ampelovirus species, the complete genome sequence assembled from Contig-F belongs to the new subgroup I ampelovirus (Figure 2), for which, we propose the name pineapple mealybug wilt-associated virus 7 (PMWaV7) according to the current species name nomenclature. ORF1a encodes a putative polyprotein of 219 kDa with the domains L-Pro, Mtr and Hel; ORF1b encodes a putative RdRp of 53.8 kDa; ORFs 2 and 3 encode putative proteins of 3.3 kDa (p3) and 4.6 kDa (p5), respectively; ORF4, 5, 6 and 7 encodes a putative HSP70h (60.2 kDa), p57 (56.8 kDa), CP (34.3 kDa) and CPm (52.8 kDa), respectively (Supplementary Table S4). ORF8 and ORF9 encode putative proteins of 16.9 kDa (p20) and 26.5 kDa (p27), which are potentially involved in systemic transport and RNA silencing suppression, respectively [51]. No alkylation B (AlkB) domain was found in the replication-related proteins of the Reunion isolates of PMWaV1, PMWaV2, PMWaV3, PMWaV5, PMWaV6 and PMWaV7. No inter-species recombination was detected between the isolates of these six PMWaVs.

### 3.3. Phylogenetic Analyses

Phylogenetic analyses using RdRp, HSP70h, or CP aa sequences showed the placement of PMWaV2-RUN, PMWaV6-RUN and PMWaV7-RUN in *Ampelovirus* subgroup I and that of PMWaV1-RUN, PMWaV3-RUN and PMWaV5-RUN in subgroup II (Figure 2).

**Figure 3:**
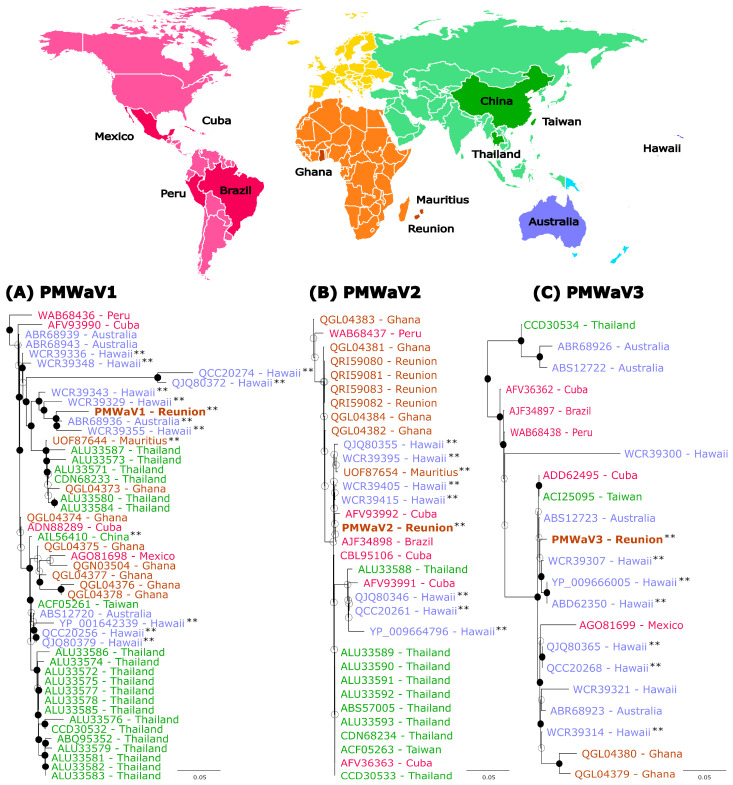
Maximum-likelihood phylogenetic trees built from the comparison of HSP70h amino acid sequences of pineapple mealybug wilt-associated virus 1 (**A**), 2 (**B**) and 3 (**C**) isolates from Reunion with sequences available from GenBank. Sequences are colored according to their geographical origin and the color legend is indicated on the map on top of the figure. Isolates from Reunion are indicated in brown and in bold. HSP70h sequences from complete genome sequences are indicated with two black asterisks. Bootstrap values equal to or greater than 70% are indicated by solid black circles. PMWaV3 (YP_0099666005), PMWaV6 (QZB90243) and PMWaV1 (YP_001642339) isolates from Hawaii were used to root the PMWaV1, 2 and 3 trees, respectively (not presented on the figures). The scale bars show the number of substitutions per site.

We took advantage of the availability in public databases of PMWaV1, PMWaV2 and PMWaV3 HSP70h sequences originating from samples collected in various locations covering most pineapple growing regions to carry out a phylogeographic analysis of the diversity of these viruses. We used 48, 32 and 21 publicly available HSP70h aa sequences for PMWaV1, PMWaV2 and PMWaV3, respectively (Figure 3). Mean sequence identities (aa) were 95.5%, 95.9% and 92.8% for the PMWaV1, PMWaV2 and PMWaV3 isolates, respectively.

There was no clear geographic structuring of the diversity, regardless of the considered *Ampelovirus* species. Sequences originating from given locations, such as PMWaV1 sequences originating from Hawaii, were sometimes scattered throughout the phylogenies and/or were more closely related to sequences originating from distant locations than to sequences originating from nearby locations. For example, the isolate PMWaV1-RUN was more closely related to isolates from Hawaii and Australia than to an isolate from nearby Mauritius (Figure 3A). Likewise, the isolate PMWaV2-RUN described in this work was grouped with highly similar sequences (minimum percentage aa identity of 99.51%) originating from Cuba, Mauritius, Thailand, Taiwan, Brazil and Hawaii, but was more distantly related to previously described isolates from Reunion [53] (Figure 3B). It must be noted however, that none of the branch of the PMWaV2 tree showed strong bootstrap support. Lastly, the isolate PMWaV3-RUN was grouped with isolates from various locations such as Australia, Cuba, Taiwan and Hawaii (Figure 3C).

### 3.4. Comparison between the Illumina Short Reads and Nanopore Long Reads Approaches

All the reads obtained using the two HTS approaches were mapped back to the six assembled ampelovirus genomes and the coverage statistics were calculated (Table 3). Between 7235 and 198,147 short reads and 17 and 2374 long reads were mapped, depending on the virus considered. For Illumina short reads, all the genomes were entirely covered, and 97.1% to 99.8% of their length was covered at a 10× sequencing depth. PMWaV2-RUN displayed the highest mean sequencing depth (N = 1606). Due to the much lower number of reads obtained with the Nanopore long reads approach, sequencing depths were drastically lower compared to the Illumina short reads approach (Table 3). Some genomes presented with relatively large mean sequencing depths, such as those of PMWaV2-RUN (N = 77), and PMWaV1-RUN (N = 53.3). However, the genomes of PMWaV5-RUN and PMWaV6-RUN were poorly covered, with mean sequencing depths of 0.4 and 1.5, respectively.

**Figure 4:**
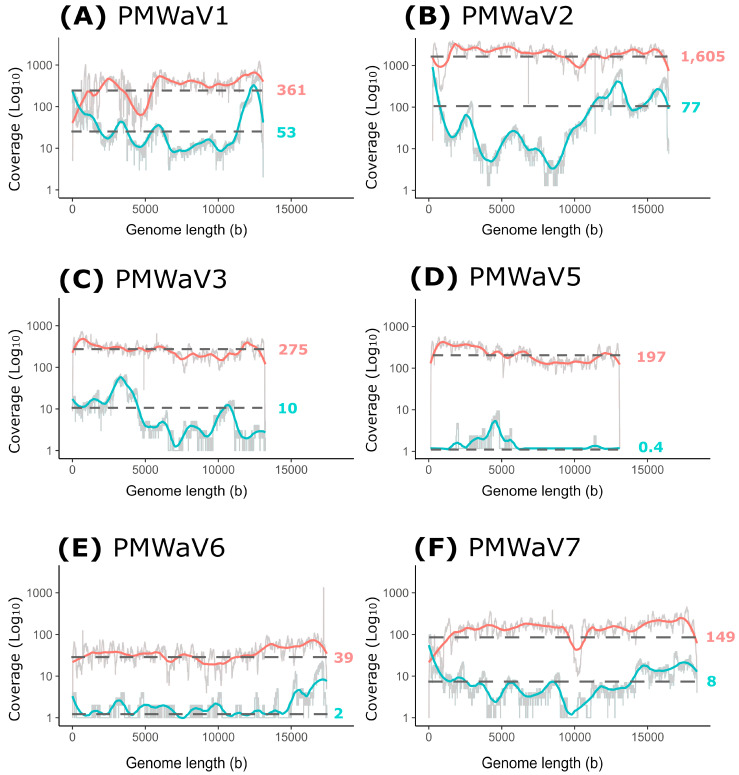
Mean sequencing depths for Illumina short reads (red curves) and Nanopore long reads (blue curves) along the genomes of PMWaV1 (**A**), PMWaV2 (**B**), PMWaV3 (**C**), PMWaV5 (**D**), PMWaV6 (**E**) and PMWaV7 (**F**). The *x*-axis represents the genomic position (in bases, b) from the 5′ end to 3′ end and the *y*-axis represents the fold coverage (Log10 scale). Colored curves were obtained after smoothing (window size of 20) of the raw depth (light grey). Mean depth values are indicated on the right of each curve.

We further analyzed the sequencing depths of Illumina short reads and Nanopore long reads along the genomes of PMWaV1, 2, 3, 5, 6 and 7 (Figure 4).

The sequencing depths for Illumina short reads was relatively stable along genome sequences (ranging from 39 to 1605), except for PMWaV1 and, to a lesser extent, PMWaV7 (Figure 4). On the contrary, large variations in coverage were observed for Nanopore long reads along the genomes of all six studied viruses (ranging from 0.4 to 77), with relatively higher depths for the 5′ and 3′ ends of the genomes, except for PMWaV3 and PMWaV5 (Figure 4D). Indeed, seven of the twelve genome extremities sequences were obtained (5′ end essentially) using Nanopore long reads sequencing (from 1 to 108 reads covering the 5′ and 3′ ends), whereas three of these 12 genome extremities sequences were obtained using Illumina short reads (from 5 to 254 reads covering the 5′ and 3′ ends). Very few reads were obtained for PMWaV5, and none could be mapped to the extremities of its genome using either Nanopore long or Illumina short reads sequencing.

Regarding the proportion of viral bases in relation to the total number of sequenced bases, important differences were observed between viral genomes (Table 3). For Illumina short reads, this proportion ranged between 34.4 bases per million of sequenced bases for PMWaV6 and 1292.0 for PMWaV2. For Nanopore long reads, it ranged between 1.6 for PMWaV5 and 373.5 for PMWaV2. If these proportions were considered proxies of the viral loads, PMWaV2 would be the virus with the highest viral load in the sampled plant, and PMWaV5 and PMWaV6 the viruses with the lowest viral loads.

**Figure 5:**
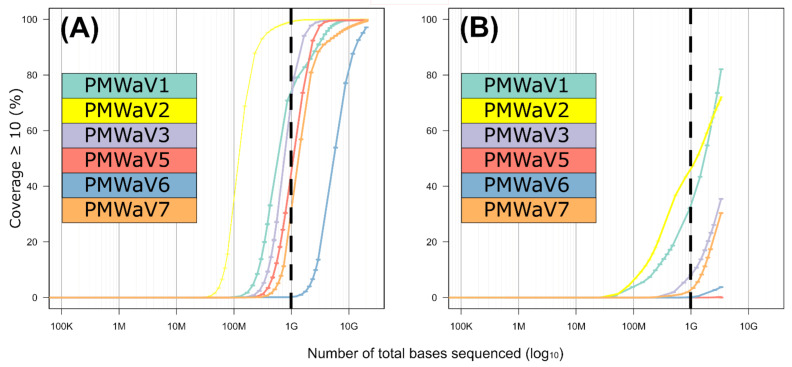
Intrapolation of sequence coverage (10×, *y*-axis) for each PMWaV for the Illumina short (**A**) and Nanopore long (**B**) reads approaches according to the number of sequenced bases (*x*-axis in Log10 scale).

In order to evaluate the correlation between the sequencing effort (i.e., the amount of raw sequences obtained per sample) and genome coverage, we calculated the sequencing depth (i.e., the number of times a nucleotide is read during sequencing) and breadth (i.e., the proportion of nucleotide positions in the consensus sequence relative to the length of the complete genome sequence) for sets of subsampled reads (Figure 5A,B). We obtained the distribution of the expected genome coverage (*y*-axis on Figure 5A,B) for every virus and for increasing sequencing efforts (*x*-axis on Figure 5A,B). A total of 15.2 Gb of sequenced data for the Illumina short reads, corresponding to the overall number of bases obtained through this approach after quality control, revealed that all ampelovirus genomes sequences exhibited an almost complete 10× depth (a minimum of 97% for PMWaV6), but a decrease in coverage breadth was observed when reducing the sequencing effort (Figure 5). Importantly, the slope of the curve (representing the rate at which bases are covered when increasing the number of reads) was mostly similar for all six viruses (Figure 5A). For a sequencing effort of 1 Gb, corresponding to a common Illumina run in which 96 samples would have been multiplexed, 10× sequencing depth would range between 29.3% and 98.6% for all viral genomes except PMWaV6. Although not all genome regions would be properly covered, at least five of the viral genomes would have been detected (with an estimation of ~1200 to 13,000 minimum reads for all of the six species). Only PMWaV6 may remain less detected, with only ~475 reads expected at 1 Gb. For this virus, the intrapolation curve showed that the breadth of coverage at a 10× sequencing depth reached 50% of the genome for a 4.7 Gb sequencing effort (Figure 5A).

For the Nanopore long reads approach, a 3 Gb sequencing effort corresponding to the total number of sequenced bases obtained after quality control when combining the two runs, did not allow the complete sequencing of any viral genome and resulted in a 10× sequencing depth for 0 to 82% of the nucleotide positions depending on the virus considered (Figure 5B). Very few reads were obtained for PMWaV5 (N = 17), for which no position was covered with a 10× depth. Contrary to the results obtained using the Illumina short reads, the slopes of the curves differed slightly between viruses (Figure 5B), suggesting that some genomes were preferentially sequenced over others. If the sequencing effort was reduced to 1 Gb, the proportion of the viral genomes sequenced at a 10× depth would be drastically reduced: no position of the genomes of PMWaV5 and PMWaV6 would be covered with a 10× coverage and it would be below 10% for that of PMWaV3 and PMWaV7. PMWaV1 and PMWaV2 would display 32.9% to 47.1% genome breadths with a 10× depth, respectively. The estimated number of reads that would be obtained for each of the six viruses targeted by this study would range from ~5 to 800.

## 4. Discussion

In this study, we recovered near complete genome sequences of isolates from six ampeloviruses infecting a wilt-diseased pineapple plant in Reunion using two different HTS approaches. One sequence obtained in this study represents the first complete genome sequence of PMWaV5, and another is the complete genome sequence of a new ampelovirus, for which we propose the name pineapple wilt-associated virus 7 (PMWaV7). The remaining four viruses (PMWaV1, PMWaV2, PMWaV3 and PMWaV6) were described previously [24], and three of them (PMWaV1, PMWaV2 and PMWaV3) were previously reported in Reunion from wilt-diseased pineapple plants [53]. Our work provides the first evidence that PMWaV5, PMWaV6 and PMWaV7 isolates are also present in Reunion, adding to the complexity of the etiology of MWP in Reunion. Mixed viral infections have been repeatedly reported in pineapple. Indeed, contigs with similarities to viruses from the *Vitivirus* (family *Betaflexiviridae*, [25]), *Sadwavirus* (family *Secoviridae*, [22,23,24]) and *Badnavirus* (family *Caulimoviridae*, [21]) genera were also identified from the 16-1 pineapple sample used in this work, providing evidence that this plant was infected by at least nine viruses from four distinct families. Vegetative propagated crops such as pineapple are known to accumulate viruses [17,24,25,54] because they do not undergo sexual reproduction, which acts as natural sanitation against viruses since the majority of plant viruses are not seed-transmitted [55]. Co-infections by large numbers of viruses may favour synergistic effects, which in the case of pineapple, could influence the severity of MWP symptoms. 

Pineapple was disseminated throughout the tropics and subtropics from its South American centre of origin (Paraguay, southern Brazil and northern Argentina) in less than 600 years through navigation routes [56,57,58], likely resulting in the spread of pineapple pests and diseases worldwide. Further intensification of plant material exchanges may also have contributed to the spread of these pests and pathogens. Indeed, the phylogeographic analyses reported in this work did not provide evidence for a geographical structuring of the diversity of PMWaV1, PMWaV2 and PMWaV3 isolates, supporting the hypothesis that PMWaVs were mainly disseminated through recurrent exchanges of infected germplasm. The analysis of a larger number of samples collected worldwide, including ancient samples conserved in herbaria throughout the world, could help refine this scenario and better understand the migration routes of PMWaVs and other pineapple viruses. A similar approach proved successful for unravelling the origin of viruses of grasses [59], grapevine [60] and cassava [61].

Our previous studies on PMWaVs [18,24,62,63] highlighted the lack of knowledge about the diversity of viruses infecting pineapple and the potential of HTS to help fill this gap. In this study, we used a combination of HTS Illumina short and Nanopore long reads to tackle this issue. Whereas the first approach requires access to Illumina sequencing devices usually affordable for medium to large laboratories, the second uses the Oxford Nanopore MinION sequencing device, which is easy to implement even in small research facilities with average levels of equipment. A probable limitation of the Nanopore long reads sequencing approach described in this work lies in the use of a cDNA-PCR barcoding kit designed for the sequencing of polyadenylated RNAs. Although this approach is suitable for mRNA sequencing, it is not suitable for not-polyadenylated RNA, such as those of the ampelovirus genomes. The sequencing was nevertheless effective because of the priming of poly-T primer in A-rich regions of ampelovirus genomes, although probably not as much as with polyadenylated RNA templates, resulting in the observed reduced efficiency of the Nanopore long reads approach. The use of random priming for cDNA synthesis could be a more efficient option for ampelovirus sequencing and viruses’ discovery in general. Nevertheless, the combination of the Illumina short reads and Nanopore long reads approaches enabled the assembly of complete or near complete genomes of PMWaV1, PMWaV2, PMWaV3, PMWaV5, PMWaV6 and PMWaV7, including the sequences of most 5′ ends and some of the 3′ ends of these viral genomes without having to resort to time-consuming RACE PCRs. Therefore, the approach described in this paper paves the way for the development of HTS-based viral indexing and sequencing of complete viral genomes, for which technical and management guidelines have been produced to cover the process of implementing HTS technologies in a research or diagnostic laboratory (selection, development, verification and validation) to detect plant pathogens and pests [64].

## 5. Conclusions

Our results underscore the existence of important viral diversity among ampeloviruses infecting pineapple in Reunion. Additional work is now required to assess the biology of these viruses, their role in the etiology of MWP, implement better risk assessment and to design appropriate disease management methods for the control of MWP [65].

## Figures and Tables

**Table 1:** Characteristics of the contigs with similarities to ampeloviruses.

Supercontigs ID (Length bp)	Number of Contigs	Contigs Length (bp)	Number of Reads	Cov ≥ 10 ^1^ (%)	Cov ≥ 100 ^1^ (%)	Mean Depth	Most Similar Virus on NCBI (BLASTn)	Accession Numbers	Identity (%) ^2^	Query Cover (%)	Virus Specises
Contig-A (13,074)	67	86–13,073	42,491	99.9	98.3	415	Pineapple mealybug wilt-associated virus 1	OP860292	86.3	99.3	PMWaV1
Contig-B (16,199)	38	81–5816	200,516	100.0	99.9	1683	Pineapple mealybug wilt-associated virus 2	OP860299	99.4	99.2	PMWaV2
Contig-C (13,229)	59	80–5040	28,779	99.8	98.3	285	Pineapple mealybug wilt-associated virus 3	MN539274	96.3	99.4	PMWaV3
Contig-D (12,971)	8	159–6146	19,534	99.8	90.6	197	Pineapple mealybug wilt-associated virus 5	EF467920	84.1	13.1	PMWaV5
Contig-E (17,440)	4	466–7117	7304	97.5	1.1	41	Pineapple mealybug wilt-associated virus 6	OP86029	99.1	99.1	PMWaV6
Contig-F (18,388)	7	389–18,092	23,269	100.0	85.1	157	Grapevine leafroll-associated virus 3	KY073324	79.3	0.5	PMWaV7

^1^ Percent genome coverage at 10X and 100X. ^2^ E value was always of 0 except for PMWaV7 with 8 × 10^-7^.

**Table 2:** Pairwise nucleotide and amino acid sequence identities in the RNA-dependant RNA polymerase (RdRp); heat shock protein 70 homolog (HSP70h); coat protein (CP) of PMWaV1, PMWaV2, PMWaV3, PMWaV5, PMWaV6 and PMWaV7 isolates whose genome were sequenced in this work; and the most closely related ampeloviruses.

Protein	Supercontig	Virus Species	Most Closely Related Virus (BLASTp)	Nucleotides Identities (%)	Amino Acid Identities (%) ¹
**RdRp**	**Contig-A**	PMWaV1	NC_010178—Pineapple mealybug wilt-associated virus 1	90.6	90.7
	**Contig-B**	PMWaV2	NC_043105—Pineapple mealybug wilt-associated virus 2	98.9	98.4
	**Contig-C**	PMWaV3	NC_043406—Pineapple mealybug wilt-associated virus 3	97.6	98.6
	**Contig-D**	PMWaV5	EF467922—Pineapple mealybug wilt-associated virus 5	85.6	85.7
	**Contig-E**	PMWaV6	MW269512—Pineapple mealybug wilt-associated virus 6	99.5	98.8
	**Contig-F**	PMWaV7	NC_004667—Grapevine leafroll-associated virus 3	55.2	**43.0**
**HSP70h**	**Contig-A**	PMWaV1	NC_010178—Pineapple mealybug wilt-associated virus 1	88.9	86.4
	**Contig-B**	PMWaV2	NC_043105—Pineapple mealybug wilt-associated virus 2	97.5	95.9
	**Contig-C**	PMWaV3	NC_043406—Pineapple mealybug wilt-associated virus 3	97.2	94.8
	**Contig-D**	PMWaV5	EF467920—Pineapple mealybug wilt-associated virus 5	84.7	91.8
	**Contig-E**	PMWaV6	MW269512—Pineapple mealybug wilt-associated virus 6	99.3	99.1
	**Contig-F**	PMWaV7	NC_004667—Grapevine leafroll-associated virus 3	55.2	**41.2**
**CP**	**Contig-A**	PMWaV1	NC_010178—Pineapple mealybug wilt-associated virus 1	91.1	94.9
	**Contig-B**	PMWaV2	NC_043105—Pineapple mealybug wilt-associated virus 2	99.5	99.2
	**Contig-C**	PMWaV3	NC_043406—Pineapple mealybug wilt-associated virus 3	97.2	97.3
	**Contig-D**	PMWaV5	NC_043406—Pineapple mealybug wilt-associated virus 3	66.6	**60.6**
	**Contig-E**	PMWaV6	MW269512—Pineapple mealybug wilt-associated virus 6	99.4	98.6
	**Contig-F**	PMWaV7	NC_022072—Blackberry vein banding-associated virus	46.9	**34.3**

**¹** Values below the current ICTV species discrimination criteria (<75% aa identity) are shown in bold.

**Table 3:** Reads mapping statistics for Illumina short and Nanopore long reads approaches (after quality control).

Virus Species	Approach	Number of Reads	Cov ≥ 1 ^1^ (%)	Cov ≥ 10 ^1^ (%)	Mean Depth	Number Viral Bases/ Million Sequenced Bases
PMWaV1	Illumina short reads Nanopore long reads	41,417 1074	100 100	99.8 82.1	361.0 53.3	310.6 234.7
PMWaV2	Illumina short reads Nanopore long reads	198,147 2374	100 99.7	100 72.0	1606 77.0	1710.1 420
PMWaV3	Illumina short reads Nanopore long reads	28,561 218	100 94.8	99.7 35.5	275.0 10.1	238.9 45.3
PMWaV5	Illumina short reads Nanopore long reads	19,517 17	99.9 18.8	99.8 0.0	197.0 0.4	168.1 1.8
PMWaV6	Illumina short reads Nanopore long reads	7235 69	99.9 53.9	97.1 3.7	39.4 1.5	45.2 8.7
PMWaV7	Illumina short reads Nanopore long reads	22,937 332	99.9 94.0	99.7 30.4	148.8 8.3	179.9 51.7

^1^ Percent genome covertage at 1X and 10X.

## Data Availability

New sequences described in this paper were deposited on GenBank under accession numbers OQ850037-OQ850042.

## Supplementary Materials

The following supporting information can be downloaded at: https://www.mdpi.com/article/10.3390/v16071146/s1. Table S1: Sequence of the primers used in RT-PCR assays for the detection of PMWaVs. Table S2: Identification of the 5′ and 3′ ends of the genome sequences of PMWaV species using RACE PCR and Nanopore long reads approaches. Table S3: Read mapping statistics for Illumina short and Nanopore long reads approaches for contigs other than *Ampeloviruses*. Table S4: Genomic organization of PMWaV sequences described on pineapple in Reunion. Table S5: List of sequences used for pairwise identity comparisons and phylogenetic analysis. (Ref. [66] is cited in Supplementary Materials Table S1).
